# Case of Intussusception of the Bowel

**Published:** 1844-07-27

**Authors:** M. W. Wilson

**Affiliations:** Schenectady, N. Y.


### THE MEDICAL EXAMINER,

### And Record of Medical Science.

Vol. VII.] PHILADELPHIA, SATURDAY, JULY 27, 1844. [No. 15.

### CASE OF INTUSSUSCEPTION OF THE BOWEL, BY M. W. WILSON, M. D. OF SCHENECTADY, N. Y.

T. E. æt. 5 years, complained of feeling unwell, on the 19th and 20th of June, 1843, and his aunt, with whom he lived, administered a dose of sulphate of magnesia, on each of these days, which operated gently on his bowels. After this his health improved until the 22d, when he was taken with fever and pain in his abdomen; tongue white and coated, and all the symptoms that usually attended the "influenza," that was then prevalent throughout the country. He was directed a purge of calomel and rhubarb, and if no action on the bowels followed, to give a common injection at the end of four or five hours. Fomentations were also directed to the abdomen. In the evening the pulse was 120 p. m.; tongue slightly coated, and the papillæ projecting near the edge. The skin was hot and dry; much pain and tenderness of abdomen. The bowels had not been moved. Directed to continue the sinapism and give an injection of warm soap-suds. This, though repeated frequently, did not succeed in emptying the bowels; the injection came from him in about fifteen minutes without fæces. The abdomen was now (three hours after the injection) very much distended. A powder composed of a grain of calomel and half a grain of ipecacuanha was directed every two hours.

23d. No change of importance in the symptoms. The powders caused some vomiting. He was directed a warm bath, to repeat the injection, and to continue the sinapisms and powders. In the afternoon he had two or three *small* discharges from his bowels which had the appearance of bloody serum. The color was dark and the odor very offensive. Pulse was 130 p. m.; tongue dark and coated; some pain (not severe) of abdomen; more restless.

24th. Expression anxious; abdomen tumid and hard. No other change in the symptoms. He has now directed half a drop of croton oil every hour until ten drops were taken, but without effect. In the evening a long injection tube was introduced in the rectum and a large quantity of tepid water thrown up, which passed from him without fæces.

25th. Vomiting of stercoraceous matter, great prostration. This circumstance confirmed a probable diagnosis that had been made of intussusception, and we proceeded at once to inflate the bowels, which was accomplished without difficulty, but without beneficial effect. A good injection tube was now introduced through the rectum in the colon and a large quantity of tepid water was thrown up with the view to reduce the invagination by the pressure of the water from below, but it was equally fruitless.

26th. Abdomen enlarged, and tympanitic, complained of less pain, great prostration. Croton oil was again administered but without result.

27th. Great prostration, vomiting continues; loss of sight. He died during the night.

*Necroscopy ten hours after death.*

On opening the abdomen a small quantity of dark coloured fluid was found in the cavity of the peritoneum. The stomach was healthy except near the cardiac orifice where there was a patch of bright red injection. The duodenum and jejunum were but slightly altered. The ileum about two feet above the ileo-cœcal valve was of a dark mahogany colour, and intussusception of about five inches was found at this place. The coats of the intestines when they came together in the intussusception were quite firmly adherent, and also of a dark mahogany colour. About a foot above the invagination a rupture of the gut had occurred, and a black tenacious mass passing from the intestine to the cavity of the abdomen blocked up the opening. This opening could hardly be caused by the dissection, for aside from the care that was practiced in the examination, the opening appeared as though caused by a different agent than the scalpel. The ileo-cœcal valve was healthy and the colon entirely empty and much contracted.

